# Dental Treatment of a Child Suffering from Non-bullous Congenital Ichthyosiform Erythroderma under General Anesthesia

**DOI:** 10.5005/jp-journals-10005-1305

**Published:** 2015-08-11

**Authors:** Rahul Choudhary, V Satish

**Affiliations:** Postgraduate, Department of Pediatric Dentistry, Jaipur Dental College Jaipur, Rajasthan, India; Associate Professor, Department of Pedodontics, College of Dentistry, Gizan University, Kingdom of Saudi Arabia

**Keywords:** Congenital, Non-bullous congenital ichthyosiform erythroderma, Skin lesion.

## Abstract

Non-bullous congenital ichthyosiform erythroderma (NBCIE) is an autosomal recessive form of inherited icthyosis appears as fine white scales that gradually replace collodion membrane. This case report describes management of 5 years and 11-month-old child with NBCIE suffering from early childhood caries (ECC) under general anesthesia.

**How to cite this article:** Choudhary R, Satish V. Dental Treatment of a Child Suffering from Non-bullous Congenital Ichthyosiform Erythroderma under General Anesthesia. Int J Clin Pediatr Dent 2015;8(2):157-162.

## INTRODUCTION

Neonatal non-bullous congenital ichthyosiform erythroderma (NBCIE) is an autosomal recessive form of inherited ichthyosis. There is confusion in nomenclature in the German literature, NBIE is referred as erythrodermic lamellar ichthyosis. The incidence of this disorder is about 1 in 300,000 births. Clinically, NBCIE appears as generalized erythroderma with fine white scales that gradually replace the collodion membrane. Other associations include ectropion, eclabium, scalp alopecia, decreased sweating with heat intolerance, and nail dystrophy. Treatment of NBCIE is based upon severity of the disease.^1^ In some patients, the teeth are normally developed but in others there may be defect and likely to develop caries. No changes are seen in relation to soft tissues. However, oral mucosa, status of primary and permanent teeth of children with NBCIE and treatment approaches for these children have not been investigated so far.^2,3^ Here, we report the dental treatment for a 5 years and 11 month old with NBCIE suffering from early childhood caries (ECC) and dental treatment under general anesthesia in order to restore esthetics, phonetics, masticatory efficiency and to provide psychological benefit to the patient.

## CASE REPORT

A 5 years and 11-month-old female reported to the department of pedodontics and preventive dentistry, Jaipur Dental College, Jaipur, India, for the treatment of her extensive carious and infective teeth. Patient complained of nocturnal pain in lower right and left back teeth region.

**Figs 1A to E:**
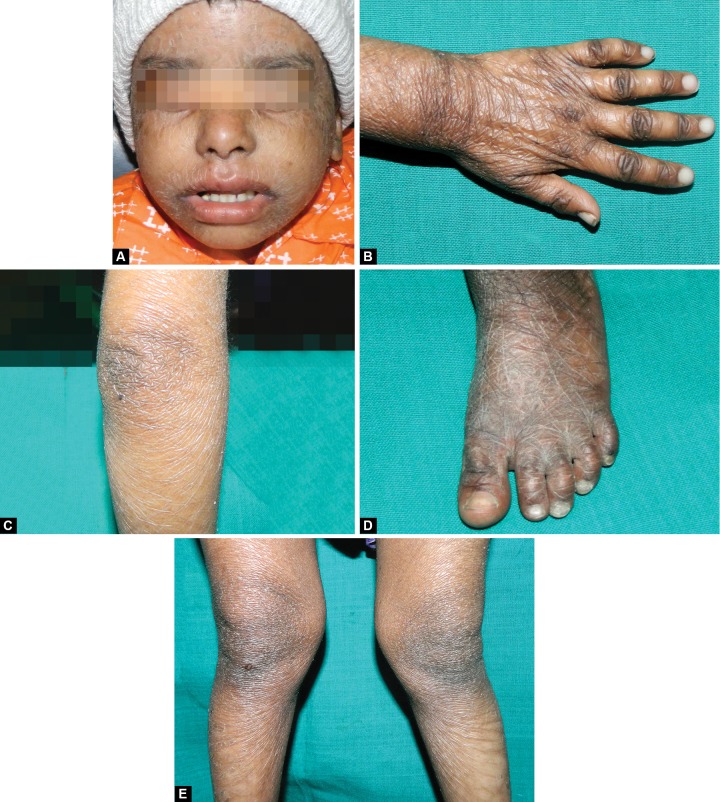
Extraoral features: (A) Face, (B) hand, (C) elbow, (D) foot and (E) knees

On medical examination, diffuse grayish-white scales present all over the body, entire face, both upper and lower limbs (Figs 1A to E). Patient had thin, dry and sparse hair and thin eyebrows. On physical examination, the gait and stature were normal. The patient was diagnosed with non-bullous ichthysiform erythroderma and was under medication since childhood and the medical reports confirmed intake of medication (syrups) from 6 months till 2 years. Later the medication was discontinued by the child’s physician due to the side effects on liver, the reports revealed enlarged size of the liver (102 mm) also called as hepatomegaly.

**Figs 2A to C:**
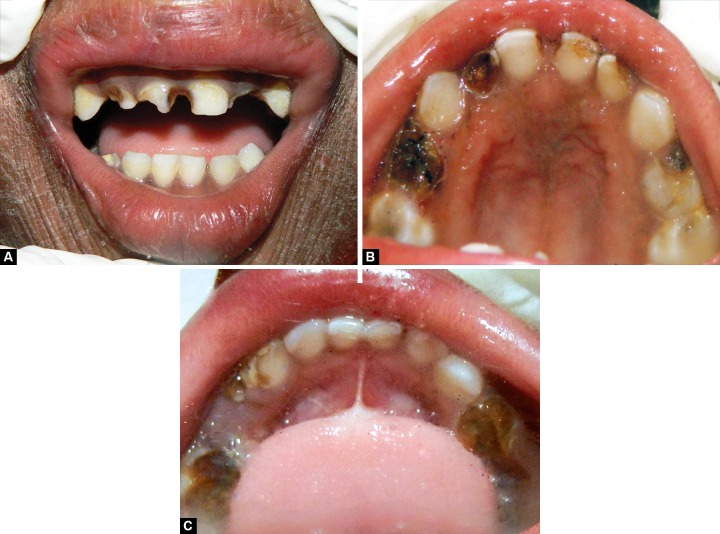
Preoperative view: (A) Maxillary anterior teeth, (B) maxillary arch and (C) mandibular arch

It was the first dental visit. The oral mucosa was normal with no remarkable findings and with no dental developmental anomalies. Patient had poor oral hygiene and teeth numbered 51, 52, 54, 61, 62, 64, 74, 75, 84, 85 were nonvital and severely destructed by caries. Remaining teeth 55, 71, 72, 73, 81, 82, 83 were vital. Disto-occlusal caries were present in 65 (Figs 2A to C). An informed consent was taken from child’s physician and dermatologist for treatment under local anesthesia but the child declaimed against the dental treatment. As the child was highly uncooperative, and with patient written consent it was decided that dental treatment will be provided under general anesthesia in a hospital setting.

## HISTOPATHOLOGY REPORT

**Fig. 3:**
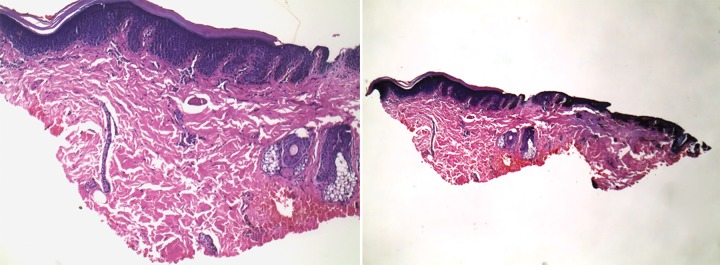
Histopathology slides

Sparse superficial perivascular lymphocytic infiltrate with mild psoriasiform hyperplasia in the epidermis. The epidermis also shows mild focal spongiosis, slight hypergranulosis and small mounds of parakeratosis amidst lamellated orhohyperkeratosis (Fig. 3).

## GENERAL ANESTHESIA

Before the treatment session, the patient was referred to cardiologist, general pediatrician and a radiologist to get medical consent for general anesthesia. Premedication injection included reglen 2.5 mg to decrease the acidity, injection succinyl choline which is a central depolarising muscle relaxant, injection glycopyrulate 0.1 mg and injection midazolam 0.5 mg.

Induction was done with IM ketamine 100 mg and maintenance was done with O_2_ + N_2_O + halothene. Reversal of anesthesia was done with physostigmine 1.25 mg along with glycopyrulate injection 0.1 mg.

## DENTAL TREATMENT

The aim of this dental treatment was to restore esthetics, chewing capacity and eliminate the infected teeth.

**Fig. 4:**
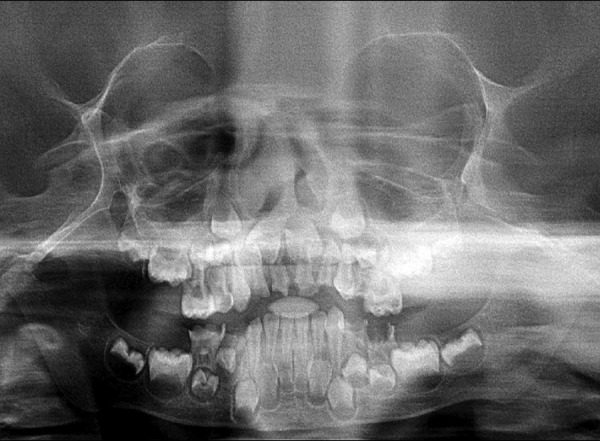
Preoperative OPG

**Figs 5A and B:**
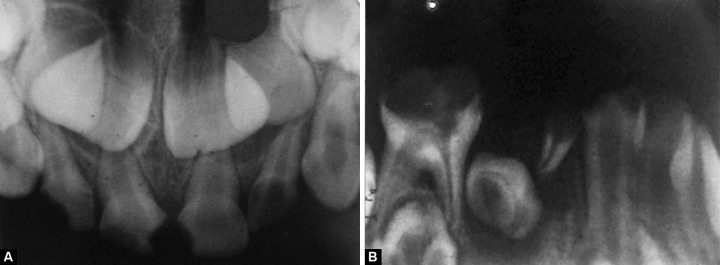
Preoperative IOPA: (A) 51, 52, 61, 62 and (B) 84,85

**Fig. 6:**
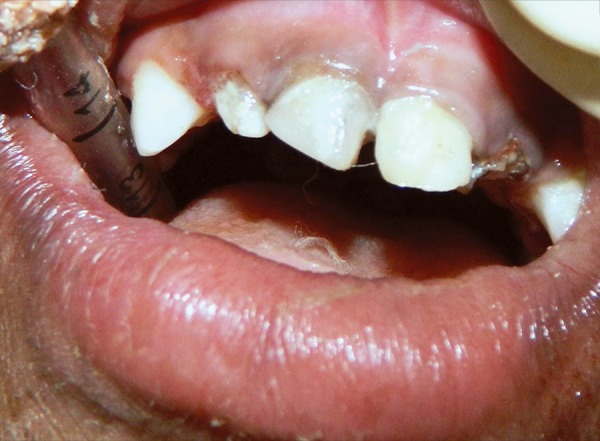
Strip crowns in relation to 51, 61

Orthopantomograph (OPG) (Fig. 4) and intraoral periapical radiographs of 51, 52, 61, 62, 85 revealed pulpal involvement (Figs 5A and B). Carious lesion was excavated and pulp was removed with K-files and H-files with frequent irrigation with saline and sodium hypochlorite. Canals were dried with paper points and obturated with calcium hydroxide and iodoform (Metapex). The teeth were restored with glass ionomer cement (KETAC MOLAR). Strip crowns (3M ESPE) were given for 51, 61 for esthetic build up (Fig. 6).

**Fig. 7:**
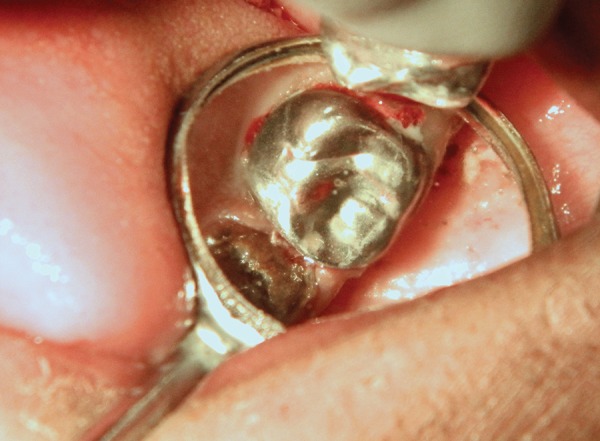
SSC in relation to 65

Carious lesion in relation to 65 was excavated using sharp spoon excavator and restored with glass ionomer cement (KETAC MOLAR). Prefabricated stainless steel crown (KIDS CROWN) was cemented on 65 (Fig. 7).

**Fig. 8:**
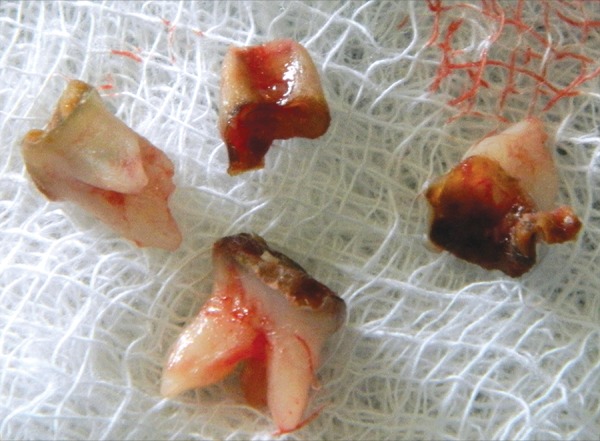
Extracted teeth

**Fig. 9:**
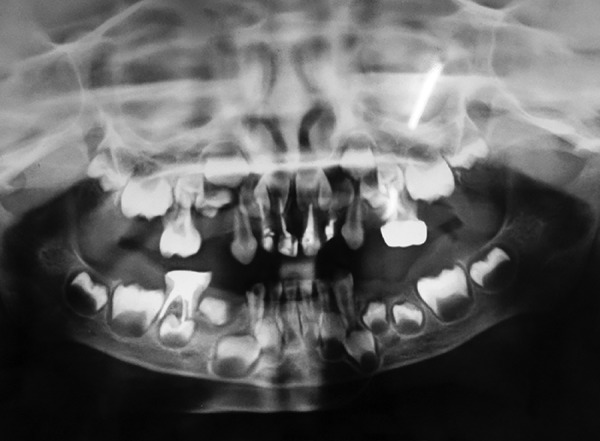
Postoperative OPG

The local anesthetic agent was injected without adrenaline as these conditions may or may not be associated with cardiac problems so to be on safer side we preferred to use local anesthetic agent without adrenaline as suggested by cardiac surgeon and anesthetist for extraction of chronically infected teeth and root stumps (54, 64, 74, 75, and 84) and alveolar sockets were compressed with sterile gauze pieces (Figs 8 and 9).

**Figs 10A to C:**
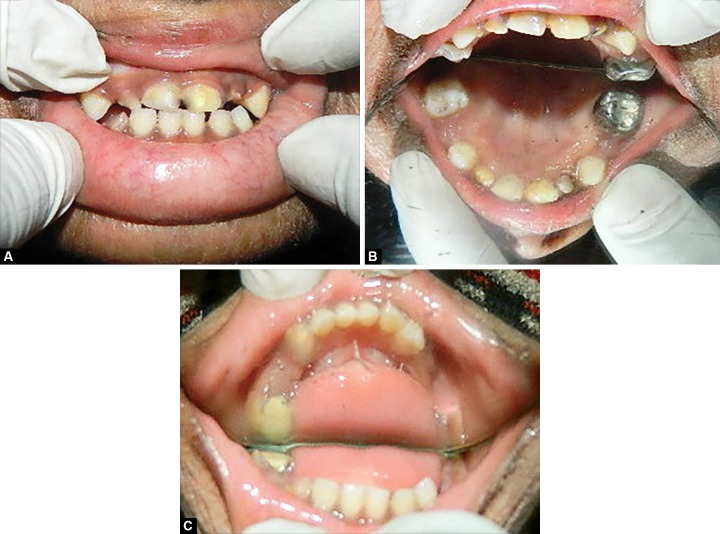
Postoperative view: (A) Maxillary anterior teeth, (B) maxillary arch and (C) mandibular arch histopathology slides

After completion of the treatments, the patient recovered uneventfully from the general anesthesia. All the dental procedures were completed without any problem. The patient was discharged after 5 hours after complete oral and general examination (Figs 10A to C).

The restorations were evaluated in terms of esthetics, phonetics and parents general satisfaction. The patient is under follow-up for the replacement of missing teeth with removable functional space maintainer. The removable functional space maintainer is planned in this case as there is multiple loss of teeth and also to restore patient’s esthetics and masticatory efficiency.

## DISCUSSION

Erythroderma is defined as an inflammatory skin disorder affecting more than 90% of the body surface.^4^ Non-bullous congenital ichthyosiform erythroderma is a rare nonblistering disorder characterized by fine grayish-white scales and erythroderma which was seen in our case. The disorder is inherited as an autosomal recessive trait except for a few cases caused by a dominant trait. An affected newborn is frequently born as a collodion baby (covered with oiled parchment-like shiny skin).^5^ Other symptoms include alopecia, deep skin fissures and nail dystrophy with or without involvement of teeth and mucosal surfaces.^6^ Hyperkeratosis is parti-cularly noticeable around the knees, elbows, and ankles.^7^

Since the 1980s, nonbullous autosomal recessive ich-thyoses have been divided into two major clinical entities, NBCIE and lamellar ichthyosis (LI).^8^ The nature of scaling and intensity of erythroderma are important clinical features that distinguish NBCIE and LI. After shedding of the collodion membrane, generalized erythroderma with fine, white or gray scales, different from the large, dark scales seen in LI.^9^ In some families with LI, transglutaminase 1 gene mutations have been identified as causative genetic defects, and transglutaminase 1 is thought to be one of the candidate molecules for NBCIE.^10^ Non-bullous congenital ichthyosiform erythroderma is unlike bullous congenital ichthyosiform erythroderma (BCIE), which is a severe autosomal dominant congenital ichthyosis which exhibits widespread blisters and erosions in the erythrodermic skin at birth.^7^

In our case, there was no developmental anomaly. The patient had early childhood caries. The reasons which could claimed to be responsible are:

- Medication for NBCIE during her early childhood. (Syrup containing sugar)^11,12^
- Poor oral hygiene.
- Increased frequency of consumption of sugar enriching foods as per patient’s given history.
  The decision to treat the child under GA was due to:
  - Definitively negative (-) behavior.
  - Frequent dental visit would have over-stretched the skin leading to damage to the skin and also discomfort to the child.

Anterior teeth after pulpectomy were restored with strip crowns to rebuild esthetics and to ensure psychological comfort.

Postoperative restoration in a pulpectomised posterior teeth was done with GIC followed by SSC in order to maintain the normal anatomy of the tooth and to restore the masticatory efficiency.

## CONCLUSION

- Children with NBCIE should undergo periodic dental visits according to ADA specification.
- Children with NBCIE are best treated under GA in order to avoid exacerbation of dermatological problems.
- Genetic counseling of the parents is advised in such cases.
- Diet counseling and oral hygiene instructions are mandatory in these patients to maintain proper oral hygiene.

## References

[1] Wang FM, Wang CC, Le CM, Lo WT (2009). Nonbullous congenital ichthyosiform erythroderma in a Neonate.. J Med Sci.

[2] Derelioglu SS, Yilmaz Y, Keles S, doi:10.1155/2013/618468. (2013). Dental treatments under General Anesthesia in a child with keratitis, ichthyosis, and deafness syndrome.. Case Rep Dent.

[3] Caceres-Rios H, Tamayo-Sanchez L, Duran-Mckinster C, De La Luz Orozco M, Ruiz-Maldonado R (1996). Keratitis, ichthyosis, and deafness (KID syndrome): review of the literature and proposal of a new terminology.. Pediatric Dermatology.

[4] Burton JL, Champion RH, Burton JL, Ebling FJG (1992). Eczema, lichenification, prurigo and erythroderma.. Rook/ Wilkinson/Ebling Textbook of Dermatology..

[5] Larregue M, Gharbi R, Daniel J, Le Marec Y, Civatte J (1976). Le bebe collodion: evolution a propos de 29 cas.. Ann Dermatol Syphiligr.

[6] Reed WB, Herwick RP, Harville D, Poster PS, Conant M (1972). Lamellar ichthyosis of the newborn: a distinct clinical entity. Its comparison to other ichthyosiform erythrodermis.. Arch Dermatot.

[7] Judge MR, Harper JI, Harper JI (1996). The ichthyoses.. Inherited Skin Diseases. The Genodermatoses..

[8] Akiyama M (1998). Severe congenital ichthyosis of the neonate.. Int J Dermatol.

[9] Williams ML, Elias PM (1985). Heterogeneity in autosomal recessive ichthyosis: clinical and biochemical differentiation of lamellar ichthyosis and nonbullous congential ichthyosiform erythroderma.. Arch Dermatol.

[10] Akiyama M (1999). The pathogenesis of severe congenital ichthyosis.. J Dermatol Sci.

[11] Bigeard L (2000). The role of medication and sugars in pediatric dental patients.. Dental Clinics of North America.

[12] Feigal RJ, Jensen ME, Mensing CA (1981). Dental caries potential of liquid medications.. Pediatrics.

